# Anandamide Influences Interleukin-1β Synthesis and *IL-1* System Gene Expressions in the Ovine Hypothalamus during Endo-Toxin-Induced Inflammation

**DOI:** 10.3390/ani11020484

**Published:** 2021-02-12

**Authors:** Monika Tomczyk, Dorota Tomaszewska-Zaremba, Joanna Bochenek, Anna Herman, Andrzej P. Herman

**Affiliations:** 1 Department of Genetic Engineering, The Kielanowski Institute of Animal Physiology and Nutrition, Polish Academy of Sciences, 05-110 Jabłonna, Poland; m.tomczyk@ifzz.pl (M.T.); jb.tst@wp.pl (J.B.); 2 Department of Animal Physiology, The Kielanowski Institute of Animal Physiology and Nutrition, Polish Academy of Sciences, 05-110 Jabłonna, Poland; d.tomaszewska@ifzz.pl; 3 Faculty of Health Sciences, Warsaw School of Engineering and Health, 02-366 Warsaw, Poland; anna.herman@onet.pl

**Keywords:** anandamide, endocanabinoids, inflammation, endotoxin, LPS, IL-1β, hypothalamus, central nervous system

## Abstract

**Simple Summary:**

Pro-inflammatory cytokines are considered to be one of the most important mediators affecting the function of central nervous system during an immune/inflammatory challenge. It was found that in acting on different hypothalamic nuclei, pro-inflammatory cytokines influence the centrally regulated processes including reproduction. Recently, it has been shown that the endocannabinoid system and endogenous cannabinoids may attenuate the inflammatory response. Therefore, in our study we examined the influence of anandamide, one of the earliest known endocannabinoids, on the synthesis of interleukin (IL)-1β and *IL-1* system gene expressions in the hypothalamic structures involved in gonadotropin-releasing hormone (GnRH)-ergic activity, and thus the central control of reproduction, during immune stress induced by endotoxin injection. It was found that anandamide inhibited lipopolysaccharide (LPS)-stimulated synthesis of IL-1β in the hypothalamus, likely affecting posttranscriptional levels of this cytokine synthesis. Anti-inflammatory effect of anandamide at the level of central nervous system might also result from its stimulating action on IL-1 antagonist and IL-1 type II receptor gene expression. This study suggests the potential of endocannabinoids and/or their metabolites in the inhibition of inflammatory process at the level of the central nervous system, as well as their usefulness in the therapy of inflammation-induced neuroendocrine disorders, but further detailed research is required to investigate this issue.

**Abstract:**

This study evaluated the effect of anandamide (AEA) on interleukin (IL)-1β synthesis and gene expression of *IL-1β*, its type I (*IL-1R1*) and II (*IL-1R2*) receptors, and IL-1 receptor antagonist (*IL-1RN*) in the hypothalamic structures, involved in the central control of reproduction, during inflammation. Animals were intravenously (i.v.) injected with bacterial endotoxin-lipopolysaccharide (LPS) (400 ng/kg) or saline, and two hours after LPS administration., a third group received i.v. injection of AEA (10 μg/kg). Ewes were euthanized one hour later. AEA injection (*p* < 0.05) suppressed LPS-induced expression of IL-1β protein in the hypothalamus. The gene expression of *IL-1β*, *IL-1RN*, and *IL-1R2* in the hypothalamic structures was higher (*p* < 0.05) in animals treated with both LPS and AEA in comparison to other experimental groups. AEA administration did not influence LPS-stimulated *IL-1R1* gene expression. Our study shows that AEA suppressed IL-1β synthesis in the hypothalamus, likely affecting posttranscriptional levels of this cytokine synthesis. However, anti-inflammatory effect of AEA might also result from its stimulating action on *IL-1RN* and *IL-1R2* gene expression. These results indicate the potential of endocannabinoids and/or their metabolites in the inhibition of inflammatory process at the level of central nervous system, and therefore their usefulness in the therapy of inflammation-induced neuroendocrine disorders.

## 1. Introduction

During inflammation induced by viral or bacterial infection, a number of immune and non-immune cells produce cytokines and other inflammatory mediators able to affect physiological processes in the organism, including those occurring in the central nervous system (CNS) [1]. Acting on different hypothalamic nuclei, pro-inflammatory cytokines may influence the centrally regulated processes such as food intake, thermogenesis, rest/activity circadian rhythm, and reproduction [2,3]. Both acute and prolonged inflammations decrease the circulating concentration of luteinizing hormone (LH) and therefore affect the reproductive process [4,5]. These changes are connected mainly with decreased gonadotropin-releasing hormone (GnRH) secretion in the hypothalamus [6,7,8,9]. It is worth mentioning that in sheep, which were used as an animal model in the present study, GnRH neurons do not form dense clusters but are spread from the brain septum and the diagonal band of Broca through the preoptic area (POA) to the anterior hypothalamus (AHA) and the medial basal hypothalamus (MBH). Nonetheless, more than a half of the population of GnRH-ergic neurons in the hypothalamus have perikarya located in the POA [10]. The inflammation caused by numerous physiological pathways impede GnRH/LH secretion, however, centrally acting pro-inflammatory cytokine-interleukin (IL)-1β is thought to be among the most important mediators inhibiting reproductive processes during the immune challenge [1,11,12]. During inflammatory stimuli, IL-1β is produced at the periphery by specific cells, mainly monocytes and macrophages and numerous other cells including lymphocytes T and B, microglia cells, endothelial cells, astrocytes, oligodendrocytes, and neurons [13,14]. It was previously shown that peripheral cytokines (e.g., IL-1β) are able to reach the CNS by crossing the fenestrated capillaries in the structures such as the choroid plexus (CP), median eminence, or organum vasculosum of the lamina terminalis [11]. At the period of inflammation, these cytokines may also reach the brain via the blood–brain barrier by saturated, self-inhibitable transport mechanisms [15]. Moreover, in recent studies it was shown that during inflammation activated cells of the CP might express and release pro-inflammatory cytokines into the cerebrospinal fluid (CSF) [16,17,18]. However, the local synthesis of centrally acting inflammatory cytokines in the CNS is another important source of such proteins. In numerous studies, it was observed that pro-inflammatory interleukins, including IL-1β, are synthetized directly in the hypothalamus [4,14,19,20]. The ability of IL-1β to impair secretion of GnRH enables the presence of interleukin 1 receptor (IL-1R1) in the hypothalamic structures involved in GnRH-ergic activity [14,21]. Moreover, the expression of *IL-1Rs* was demonstrated directly on GnRH neurons. This fact proves that IL-1β ligands could take part in the modulation of these cells’ activity [22].

Recently, the majority of scientific research has been focused on the importance of the endocannabinoid system and endogenous cannabinoids. It was found that cannabinoids acting through their corresponding receptors are able to attenuate the inflammatory response [23,24]. Anandamide (AEA) is the earliest known endocannabinoid that takes part in the immune system regulation [25,26]. It may influence various physiological processes acting through two major cannabinoid receptors: type 1 (CB1) and 2 (CB2). CB2, also known as “spleen type”, is predominantly expressed by the immune cells but also, to a lesser extent, in the brain [24,27]. CB1 mainly occurs in the CNS; it has been detected in the cerebral cortex, limbic system structures, cerebellum, pituitary gland, and above all else in the hypothalamus [28]. Having in mind the fact that on immune cells both CB1 and CB2 receptors have been detected, it could be suggested that cannabinoids play a substantial role in the immune system regulation. It was demonstrated that administration of delta9-tetrahydrocannabinol (THC) into mice generated a noticeable apoptosis in T and dendritic cells. Cannabinoids may downregulate the production of cytokine and chemokine and, as presented in some models, may upregulate T-regulatory cells (Tregs) in order to suppress inflammatory responses [29]. From this point of view, cannabinoids may be considered as a potent treatment against inflammatory disorders. It is worth mentioning, that in our pilot study on ewes, the lack of effect of single AEA injection on the *IL-1β* and *IL-1RN* expression in the hypothalamus was found.

Therefore, the aim of our study was to check whether peripheral administration of AEA will influence the expression of IL-1β protein and mRNA encoding IL-1β, its type I and II receptors, and interleukin 1 receptor antagonist (IL-1RN) in the hypothalamic structures involved in GnRH-ergic activity in ewe during inflammation induced by injection of a lipopolysaccharide (LPS)—bacterial endotoxin present in the outer membrane of Gram-negative bacteria.

## 2. Materials and Methods

### 2.1. Animals and Experimental Design

The study was performed on 18 adult 6-year-old Blackhead ewes during the reproductive season (from September to October). The animal acclimation to the experimental conditions was 1 month before the experiment. Ewes body condition score was kept at an estimated level of 3 points on a 5-point scale. The animal housing system was customized for sheep requirements, according to regulations of animal welfare. Each ewe was kept in individual pen in the fold, and in order to prevent separation stress, we gave them visual contact with other animals. The good health and condition of animals was assured by commercial concentrates with hay fed to ewes and not limited access to water, according to the recommendations of the National Research Institute of Animal Production for adult ewes [30].

The synchronization of estrous cycle stage was made by the Chronogest CR (Merck Animal Health, Boxmeer, the Netherlands) according to the procedures described in our previous studies [31,32]. Each ewe had a vaginal sponge containing 20 mg of cronolone (Chronogest CR; Merck Animal Health, Boxmeer, the Netherlands) placed for 14 days. After the sponge removal, animals were intramuscularly injected with 500 IU pregnant mare’s serum gonadotropin (PMSG; Folligon; Merck Animal Health, Boxmeer, the Netherlands). The experimental procedures started 7 days following PMSG injection, in luteal phase of estrus cycle. An immune/inflammatory challenge was induced by the intravenous (i.v.) administration of 400 ng/kg LPS from *Escherichia coli* 055:B5 (Sigma-Aldrich, St. Louis, MO, USA), dissolved in saline (0.9% w/v NaCl) (Baxter, Deerfield, IL, USA) at a concentration of 10 mg/L.

All experimental procedures carried out on animals were performed according to the guidelines of the Local Ethics Committee of Warsaw University of Life Sciences-SGGW (authorization no. WAW2/70/2017).

### 2.2. Experimental Procedures

Venous catheters were implanted into the jugular vein on the day prior to the experiment. Ewes were randomly divided into 3 groups: control (*n* = 6), LPS-treated (*n* = 6), and treated with LPS and AEA (*n* = 6) (see Table 1). Two hours after LPS injection, the animals from the third group additionally received i.v. injection of AEA (Sigma-Aldrich, St. Louis, MO, USA) at a dose of 10 µg/kg body weight chosen on the basis of a previous study [33]. Immediately after the blood collection, ewes were euthanized, and their brains were instantaneously removed from the skulls. Then, the brain structures such as POA, AHA, and MBH were dissected and at once frozen in liquid nitrogen. Collected hypothalamic tissues were stored at −80 °C.

### 2.3. IL-1β Concentration Assesment

The concentrations of IL-1β in the hypothalamus were assayed using IL-1β ELISA kits (cat. no. E0041Sh; Bioassay Technology Laboratory, Shanghai, China) designed and validated for sheep. In the case of the ELISA assay, the hypothalamic tissues collected from each ewe were combined into 1 sample and homogenized according to the method described elsewhere [31]. The assays procedure was performed according to the instructions provided by the manufacturer. Plates incubation and absorbance measurement at wavelength of 450 nm were estimated using the VersaMax reader (Molecular Devices LLC., Sunnyvale, CA, USA). The assay sensitivity was 0.24 pg/mL. IL-1β concentration in the hypothalamus was computed relative to total protein content of the sample assayed by the Bradford method.

### 2.4. Relative Gene Expression Determination

We followed the previously published methods of Herman et al. [31]. For the total RNA isolation from the collected structures such as POA, AHA, and MBH, we used the NucleoSpin RNA kit (MACHEREY-NAGEL GmbH and Co, Düren, Germany). The concentration and purity of isolated RNA were quantified spectrophotometrically by measuring the optical density at 230, 260, and 280 nm in a NanoDrop 1000 instrument (Thermo Fisher Scientific Inc., Waltham, MA, USA). The verification of RNA integrity was carried out by electrophoresis using 1% agarose gel stained with ethidium bromide (Sigma-Aldrich, St. Louis, MO, USA). The synthesis of complementary DNA (cDNA) was performed using 1000 ng of total RNA and components of Maxima First Strand cDNA Synthesis Kit for RT-qPCR (Thermo Fisher Scientific Inc., Waltham, MA, USA).

Real-time RT-PCR analysis was carried out with the use of the HOT FIREPol EvaGreen qPCR Mix Plus (Solis BioDyne, Tartu, Estonia) and HPLC-grade oligonucleotide primers (Genomed, Warszawa, Poland) in line with the method described elsewhere [34]. Specific primers to determine the expression of the genes of interest and housekeeping genes were chosen on the basis of our previous experience (see Table 2). One reaction mixture of total volume amounting 15 μL contained 3 μL of PCR Master Mix, 10.05 μL of RNase-free water, 0.45 μL of primers (0.225 μL each primer), and 1.5 μL of the cDNA template. The PCR reactions were carried out using Rotor-Gene 6000 instrument (Qiagen, Dusseldorf, Germany) with the following protocol: 95 °C for 15 min and 30–35 cycles of 95 °C for 10 s for denaturation, 59 °C for 20 s for annealing, and 72 °C for 10 s for extension. A final melting curve analysis and agarose gel electrophoresis of PCR products were performed to verify the specificity of the amplification reaction. The relative gene expression was calculated using the comparative quantification option [35] of the Rotor Gene 6000 software 1.7. (Qiagen, Dusseldorf, Germany) with reference to the mean expression of 3 housekeeping genes: glyceraldehyde-3-phosphate dehydrogenase (*GAPDH*), β-actin (*ACTB*), and histone deacetylase1 (*HDAC1*).

### 2.5. Statistical Analysis

Statistical analysis was performed using the STATISTICA 10 software (Stat Soft. Inc., Tulsa, OK, USA). The results of gene and protein expression were analyzed using one-way analysis of variance (ANOVA) to identify the influence of anandamide on protein and gene expression during immune challenge and were followed by a post hoc Fisher’s least significance test. The results are presented as the mean ± standard error of the mean (S.E.M.); statistical significance was set at *p* < 0.05.

## 3. Results

### 3.1. Effect of AEA Injection on LPS-Induced Synthesis of IL-1β in the Hypothalamus of Endotoxin-Treated Ewes

**Figure 1:**
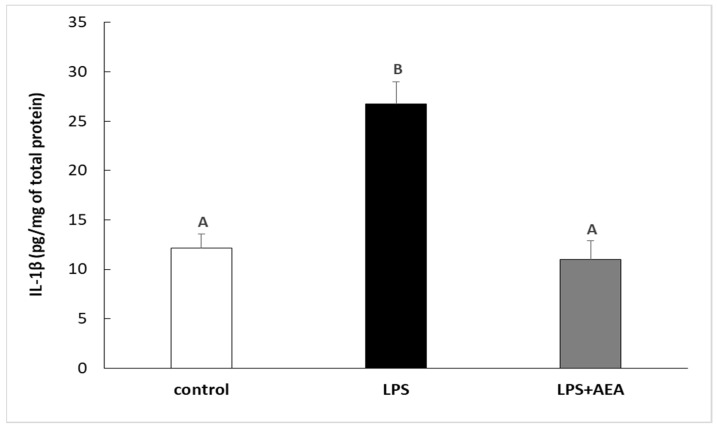
The effect of intravenous (i.v.) anandamide (AEA) injection (10 µg/kg; i.v.) and lipopolysaccharide (LPS)-induced inflammation (400 ng/mg; i.v.) on the concentration of interleukin (IL)-1β in the hypothalamus. Data are presented as the mean value ± S.E.M. Different letters indicate significant (*p* < 0.05) differences according to a one-way ANOVA followed by Fisher’s least significance test.

Injection of LPS increased (*p* < 0.05) the concentration of IL-1β protein in the hypothalamic tissue in comparison with the control group. Contrarily, the peripheral administration of AEA diminished this stimulatory effect of LPS on the expression of IL-1β protein in the ovine hypothalamus. The concentration of IL-1β in the group treated simultaneously with LPS and AEA was lower (*p* < 0.05) in comparison to the group treated only with LPS and did not differ from the IL-1β expression determined in control individuals (Figure 1).

### 3.2. Effect of AEA Injection on IL-1 Gene Expression System in the Hypothalamus of Endotoxin-Treated Ewes

**Figure 2:**
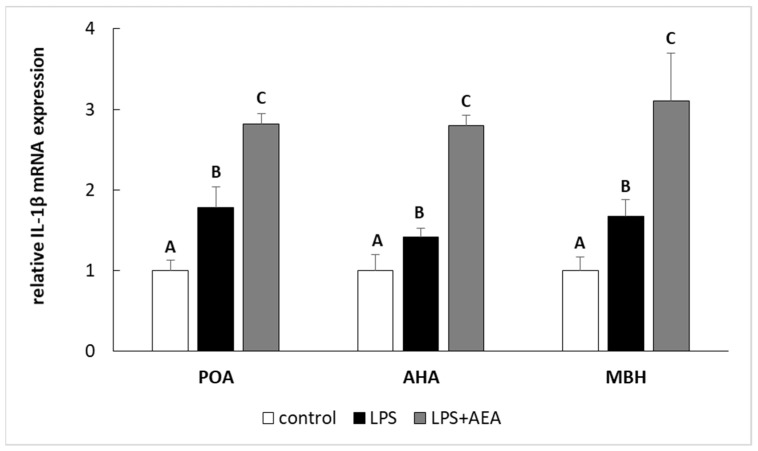
The effect of intravenous (i.v.) anandamide (AEA) injection (10 µg/kg; i.v.) and lipopolysaccharide (LPS)-induced inflammation (400 ng/mg; i.v.) on the relative interleukin (IL)-1β mRNA level in preoptic area (POA), anterior hypothalamus (AHA), and medial basal hypothalamus (MBH) normalized to the mean expression of three reference genes (*GAPDH*, *HDAC1*, *ACTB*—see Table 2.). All data are presented as the mean value ± S.E.M. Different letters indicate significant (*p* < 0.05) differences according to a one-way ANOVA followed by Fisher’s least significance test.

**Figure 3:**
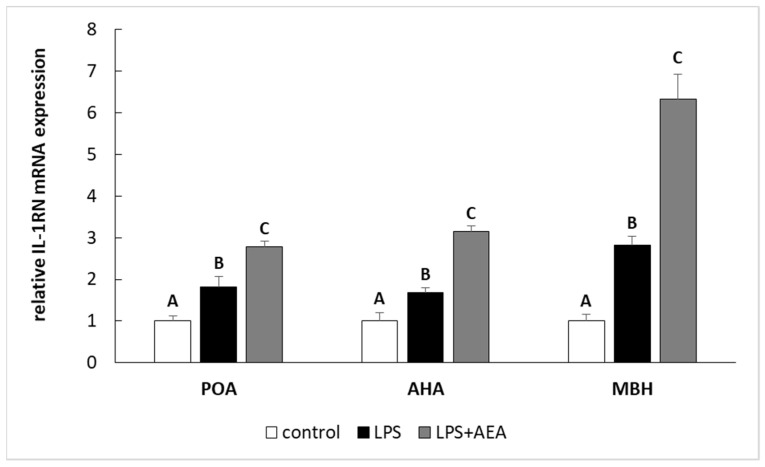
The effect of intravenous (i.v.) anandamide (AEA) injection (10 µg/kg; i.v.) and lipopolysaccharide (LPS)-induced inflammation (400 ng/mg; i.v.) on the relative interleukin 1 antagonist (IL-1RN) mRNA level in preoptic area (POA), anterior hypothalamus (AHA), and medial basal hypothalamus (MBH) normalized to the mean expression of three reference genes (*GAPDH*, *HDAC1*, *ACTB*—see Table 2). All data are presented as the mean value ± S.E.M. Different letters indicate significant (*p* < 0.05) differences according to a one-way ANOVA followed by Fisher’s least significance test.

Inflammation induced by LPS treatment increased (*p* < 0.05) the relative level of *IL-1β* and *IL-1RN* gene expression in all studied structures such as the POA, AHA, and MBH. Furthermore, in the animals simultaneously treated with LPS and AEA, the gene expression of both *IL-1β* (Figure 2) and *IL-1RN* (Figure 3) in all analyzed hypothalamic structures was higher (*p* < 0.05) compared to control and LPS-treated groups.

**Figure 4:**
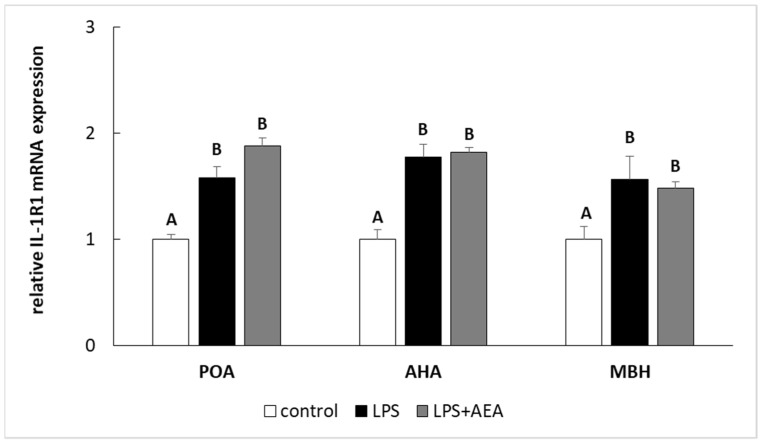
The effect of intravenous (i.v.) anandamide (AEA) injection (10 µg/kg; i.v.) and lipopolysaccharide (LPS)-induced inflammation (400 ng/mg; i.v.) on the relative receptor type I interleukin 1 (IL-1R1) mRNA level in preoptic area (POA), anterior hypothalamus (AHA), and medial basal hypothalamus (MBH) normalized to the mean expression of three reference genes (*GAPDH*, *HDAC1*, *ACTB*—see Table 2). All data are presented as the mean value ± S.E.M. Different letters indicate significant (*p* < 0.05) differences according to a one-way ANOVA followed by Fisher’s least significance test.

Endotoxin treatment elevated (*p* < 0.05) the level of *IL-1R1* gene expression in all analyzed hypothalamic structures. On the other hand, the gene expression of *IL-1R1* in these hypothalamic structures in ewe simultaneously treated with LPS and AEA did not differ in comparison to this gene expression in the endotoxin-treated group; however, it was higher (*p* < 0.05) when compared to the control animals (Figure 4).

**Figure 5:**
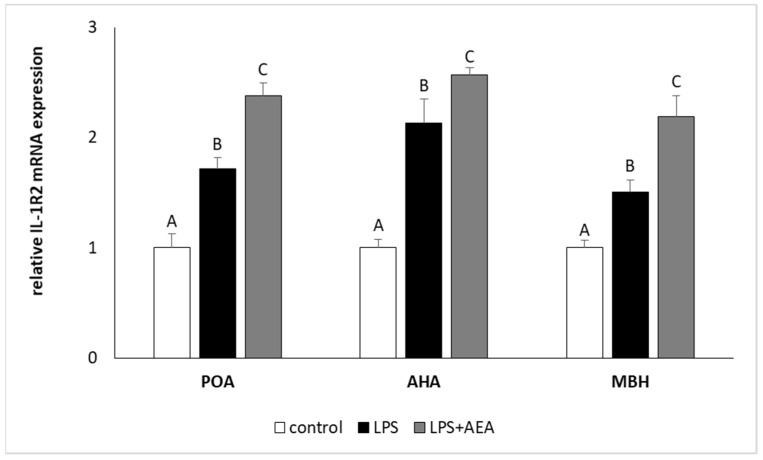
The effect of intravenous (i.v.) anandamide (AEA) injection (10 µg/kg; i.v.) and lipopolysaccharide (LPS)-induced inflammation (400 ng/mg; i.v.) on the relative receptor type II interleukin 1 (IL-1R2) mRNA level in preoptic area (POA), anterior hypothalamus (AHA), and medial basal hypothalamus (MBH) normalized to the mean expression of three reference genes (*GAPDH*, *HDAC1*, *ACTB*—see Table 2). All data are presented as the mean value ± S.E.M. Different letters indicate significant (*p* < 0.05) differences according to a one-way ANOVA followed by Fisher’s least significance test.

Injection of LPS elevated (*p* < 0.05) the level of *IL-1R2* gene expression in all studied hypothalamic structures. However, in contrast to *IL-1R1* gene expression, the simultaneous administration of LPS and AEA increased (*p* < 0.05) *IL-1R2* gene expression in the POA, AHA, and MBH in comparison to both control and LPS-treated groups (Figure 5).

## 4. Discussion

We have shown that LPS-induced inflammation stimulated the synthesis of IL-1β in the hypothalamus both at mRNA and protein levels. These results were not surprising and confirmed the conclusions achieved in other studies that showed that systemic inflammation increases the expression of IL-1β in the hypothalamus of many species including rat [37], guinea pig [38], and sheep [2,31,39,40]. It is worth mentioning that at least a partial increase in the expression of hypothalamic IL-1β could be induced by stress provoked by LPS injection because it was proved that stress condition also stimulates the synthesis of IL-1β in the hypothalamus [19]. It was also shown that LPS-induced inflammation increased *IL-1RN* gene expression in the hypothalamus. IL-1RN is a natural anti-inflammatory factor. One of its major features is high affinity binding to the IL-1R1 with no conformational changes. As a result, IL-1RN blocks the IL-1b/IL-1R1 binding. Thus, IL-1β is unable to interact with corresponding receptor, and the intracellular signal that promotes further immune stimulation does not arise [41]. Moreover, IL-1RN is a terminal anti-inflammatory factor that can not only competitively antagonize the biological effects of IL-1, but also inhibit the effects of other inflammatory factors [42]. Furthermore, it was reported that the synthesis of IL-1RN rises during various pathological states characterized by local or systemic inflammation [43]. Therefore, the increased IL-1RN synthesis in the hypothalamus during peripheral inflammation suggests that three hours after LPS, not only do inflammatory processes occur, but also anti-inflammatory mechanisms are activated in order to protect the central tissues against unrestrained development of the inflammatory response.

Endotoxin-induced inflammation also increased the gene expression of IL-1 receptor type 1 and 2 in all analyzed hypothalamic structures. This observation is consistent with the results of previous studies in which peripheral inflammation is thought to stimulate the gene expression of *IL-1R1* and *IL-1R2* in the brain [31,39,40,44]. IL-1R1 plays an important role in initiating inflammatory response. It is a signal-transmitting receptor, triggered by both IL-1α and IL-1β ligands. The IL-1R1, with its intracellular domain, is responsible for initializing the inflammatory signaling processes in target cells [45]. The activation of IL-1R1 through its agonist is required to elicit intracellular signal and further development of immune response [46]. On the other hand, IL-1R2 is a membrane-bound protein expressed on the surface of various cells such as endometrial epithelial cells, basophils, neutrophils, monocytes, and activated T and B cells but it was also found in the brain tissue. IL-1R2 inhibits pro-inflammatory IL-1 effect by acting as a decoy receptor and by preventing IL-1/IL-1R1 binding [43].

The suppressive action of AEA on the synthesis of IL-1β, reported in our study, is generally consistent with other studies that showed that endocannabinoids and cannabinoids exhibit anti-inflammatory properties. It was found that AEA treatment reduced the serum level of pro-inflammatory IL-1β in rats exposed to water immersion and restrain stress [47]. Anti-inflammatory effects of cannabinoids were also observed in the brain tissue in neurodegenerative diseases occurring with accompanied neuroinflammation. A study performed on neurodegenerative disease murine models indicated that cannabidiol decreased the level of pro-inflammatory cytokines, including IL-1β, in the central nervous system [48]. In other viral study on mice administration of R(+)WIN55,212, CB1 agonist significantly reduced the expression of mRNA encoding pro-inflammatory cytokines such as IL-1β, IL-6, and tumor necrosis factor α [49]. Moreover, in vitro studies on rat microglial cells showed that cannabinoids inhibited LPS-stimulated gene expression of pro-inflammatory cytokines including IL-1β [50]. Although many studies suggest that there is a link between cannabinoids and TLR signaling pathways that leads to the synthesis of pro-inflammatory cytokines including IL-1β [51], the definition of direct molecular mechanism still awaits further study. On the other hand, the effect of cannabinoids on the IL-1β synthesis may be more elusive. It has been reported that AEA stimulated secretion of IL-1β from RAW264.7 cells [52]. Moreover, the administration of the psychoactive component of cannabis, delta 9-tetrahydrocannabinol (THC), activated cerebellar microglia and increased the expression of neuroinflammatory markers, including IL-1β [53]. The same pro-inflammatory effect of THC was reported in studies on murine resident peritoneal macrophage cultures, showing that cannabinoid treatment increased, in a dose-dependent manner, the secretion of IL-1β [54]. The different effect of AEA on the synthesis of IL-1 at the level of mRNA and protein expression demonstrated in our research indicates that endocannabinoids may influence cytokine expression through different cellular pathways. The findings that changes in IL-1β mRNA expression did not correspond with this cytokine protein synthesis suggests that inhibitory effect of AEA is targeted on a post-transcriptional level of IL-1β synthesis. It is known that after transcription, the inactive IL-1β precursor accumulates in the cytosol until it is processed by the activation of nucleotide-binding domain and leucine-rich repeat pyrin-containing protein-3 (NLRP3) and caspase-1 into an active cytokine [55,56]. Therefore, inhibition of NLRP3 inflammasome results in the reduction of the secretion of mature IL-1β. It was previously found that AEA and its COX-2 metabolite-prostaglandin E2 ethanolamide may exert an inhibitory effect on the NLRP3 inflammasome formation and activation [57]. Moreover, it seems that the inhibitory effect of anandamide on NLRP3 inflammasome activation could be common feature of cannabinoids because recent studies on LPS-nigericin-stimulated THP-1 monocytes also showed that cannabidiol suppressed NLRP3 inflammasome activation [58]. However, it is also possible that AEA influences the expression of mature IL-1β in the hypothalamic tissue by stimulating processes leading to degradation of this cytokine. It was found that activated matrix metalloproteinase (MMP)-1, MMP-2, MMP-3, and MMP-9 are responsible for degradation of IL-1β [59]. In turn, it was reported that AEA, acting via CB1 and transient receptor potential vanilloid-1, induced production of MMP-2 [60].

Our experiment also showed that animals co-treated with LPS and AEA are characterized by higher gene expression of *IL-1RN* and *IL-1R2* but not *IL-1R1* in all hypothalamic structures involved in GnRH-ergic activity in comparison to control and LPS-treated animals. The findings that in the group treated with LPS and AEA, the gene expression of *IL-1RN* was significantly raised compared to LPS-treated and control groups suggests that the anti-inflammatory effect of AEA in the hypothalamus may result from the stimulation of IL-1RN synthesis, which in turn competes with IL-1β for binding to its receptor. This mechanism of anti-inflammatory action of cannabinoids was stated in previous in vitro studies that showed potent anti-inflammatory effect of cannabinoids in murine glial and neuronal cell cultures. It was shown that culture of murine glial and neuronal cell incubated with cannabinoid agonists during inflammatory stimuli resulted in increased concentration of IL-1RN in the analyzed supernatants. Moreover, the authors postulated that induction of endogenous IL-1RN is essential for the neuro-protective effects of CBs [61]. The fact that AEA treatment did not influence *IL-1R1* gene expression in the hypothalamus suggests that potential anti-inflammatory effect of AEA does not result from the reduced sensitivity of brain tissue to IL-1 action. However, the obtained results suggest the existence of another, novel mechanism of anti-inflammatory action of endocannabinoids in the central tissue on the basis of the increased synthesis of IL-1R2. As was mentioned above, acting as a decoy receptor IL-1R2 prevents IL-1/IL-1R1 binding, reducing the action of IL-1β. Additionally, IL-1R2 exists in both a membrane-bound and soluble form (sIL-1R2) with biological properties alike to both a decoy receptor and a binding protein [62]. Therefore, some amount of IL-1R2 from the hypothalamus may be released into the CSF, which in turn may reduce the amount of free, centrally acting IL-1β. 

## 5. Conclusions

Summarizing, our study showed that AEA interfered with the synthesis of interleukin-1β and IL-1 system gene expressions in the hypothalamic structures involved in GnRH-ergic activity during an immune/inflammatory challenge. It was found that AEA inhibited the LPS- stimulated synthesis of central IL-1β in the hypothalamus, likely affecting posttranscriptional levels of this cytokine synthesis. However, anti-inflammatory effect of anandamide at the level of central nervous system might also result from its stimulating action on *IL-1RN* and *IL-1R2* gene expression. However, further detailed research is required to investigate this issue. In sum, our study suggests the potential of endocannabinoids and/or their metabolites in the inhibition of inflammatory process at the level of central nervous system, and therefore their usefulness in the therapy of inflammatory-induced neuroendocrine disorders.

## Figures and Tables

**Table 1:** Experiment organization chart.

Group		No. of Animals	Experimental Treatment I	Dose (ng/kg)	Experimental Treatment II	Dose (µg/kg)
1	Control	6	NaCl	0	NaCl	0
2	LPS treated	6	LPS	400	NaCl	0
3	LPS + AEA i.v. treated	6	LPS	400	AEA i.v.	10
Total number of animals		18				

LPS: lipopolysaccharide; AEA: anandamide; i.v.: intravenous.

**Table 2:** The list of all genes analyzed by real-time PCR.

GenBank Acc. No.	Gene	Amplicon Size (bp)	Forward/Reverse	Sequence 5′→3′	Reference
NM_001034034	*GAPDH* (glyceraldehyde-3-phosphate dehydrogenase)	134	forward	AGAAGGCTGGGGCTCACT	[2]
			reverse	GGCATTGCTGACAATCTTGA	
U39357	*ACTB* (beta actin)	168	forward	CTTCCTTCCTGGGCATGG	[2]
			reverse	GGGCAGTGATCTCTTTCTGC	
BC108088.1	*HDAC1* (histone deacetylase1)	115	forward	CTGGGGACCTACGGGATATT	[2]
			reverse	GACATGACCGGCTTGAAAAT	
X54796.1	*IL-1B* (interleukin 1 beta)	137	forward	CAGCCGTGCAGTCAGTAAAA	[2]
			reverse	GAAGCTCATGCAGAACACCA	
NM_001206735.1	*IL-1R1* (interleukin 1 receptor, type I)	124	forward	GGGAAGGGTCCACCTGTAAC	[2]
			reverse	ACAATGCTTTCCCCAACGTA	
NM_001046210.1	*IL-1R2* (interleukin 1 receptor, type II)	161	forward	CGCCAGGCATACTCAGAAA	[36]
			reverse	GAGAACGTGGCAGCTTCTTT	
NM_001308595.1	*IL-1RN* (interleukin 1 receptor antagonist)	145	forward	AGGATCTGGGATGTCAACCA	[36]
			reverse	CATGGATCCCCAGGAACATA	

## Data Availability

The data presented in this study are available on request from the corresponding author.
